# Evaluation of deep learning models in contactless human motion detection system for next generation healthcare

**DOI:** 10.1038/s41598-022-25403-y

**Published:** 2022-12-14

**Authors:** Yukai Song, William Taylor, Yao Ge, Muhammad Usman, Muhammad Ali Imran, Qammer H. Abbasi

**Affiliations:** 1 Electrical Engineering, Columbia University, New York, 10027 USA; 2 James Watt School of Engineering, University of Glasgow, Glasgow, G12 8QQ UK; 3 School of Computing, Engineering and Built Environment, Glasgow Caledonian University, Glasgow, G4 0BA UK

**Keywords:** Quality of life, Scientific data, Computer science

## Abstract

Recent decades have witnessed the growing importance of human motion detection systems based on artificial intelligence (AI). The growing interest in human motion detection systems is the advantages of automation in the monitoring of patients remotely and giving warnings to doctors promptly. Currently, wearable devices are frequently used for human motion detection systems. However, such devices have several limitations, such as the elderly not wearing devices due to lack of comfort or forgetfulness and/or battery limitations. To overcome the problems of wearable devices, we propose an AI-driven human motion detection system (deep learning-based system) using channel state information (CSI) extracted from Radio Frequency (RF) signals. The main contribution of this paper is to improve the performance of the deep learning models through techniques, including structure modification and dimension reduction of the original data. In this work, We firstly collected the CSI data with the center frequency 5.32 GHz and implemented the structure of the basic deep learning network in our previous work. After that, we changed the basic deep learning network by increasing the depth, increasing the width, adapting some advanced network structures, and reducing dimensions. After finishing those modifications, we observed the results and analyzed how to further improve the deep learning performance of this contactless AI-enabled human motion detection system. It can be found that reducing the dimension of the original data can work better than modifying the structure of the deep learning model.

## Introduction

### Background

The development of the Internet of Things (IoT) has enabled the application of human motion detection in many scenarios, especially in healthcare^1^. The World Health Organisation (WHO) has reported that 37 million injuries are caused by falling and elderly people are at high risk of falling^2^. Numerous tragedies could have been avoided by the caregiver providing timely assistance if healthcare monitoring systems knew the patient was in danger and gave warnings in time, allowing the elderly and vulnerable people to lead more independent lives while keeping them safe through monitoring.

Human motion detection by wearable devices with an accelerometer is commonly used to build human motion health systems. A smartwatch can detect the fall of a patient and relay the information to a caregiver for help^3^ for example. However, it doesn’t work if people forget to wear or charge their devices. Using Radio Frequency (RF) signals does not require users to wear or charge devices. From the work described in Ref.^4^, we know that Channel State Information (CSI) can be used to detect human movements. CSI describes how a wireless signal propagates between an RF source and an RF receiver, and when a person performs certain actions, it impacts the CSI in a way that unique pattern changes are produced against each action. These patterns are the foundation for usage in deep learning (deep neural networks).

We adapt CSI data into different deep neural networks to find useful techniques to improve the performances. Firstly, we reproduce the deep neural network in Ref.^5^. Since deep learning model can learn representations of data with multiple levels of abstraction^6^. Increasing the depth can help the deep learning model learn more levels of data abstraction while increasing the width can extract more dimensions of data at each level of data abstraction. Convolutional Neural Network (CNN) has been proven to be easier to train and generalized better than the traditional fully connected layers^7,8^. Long Short-Term Memory Networks (LSTM) have proved to behave better than conventional Recurrent Neural Networks (RNNs) and LSTM can have great performance in learning time-series data. ResNet can transform the computation of gradients in backpropagation from multiplicative to additive, which can solve the problem of vanishing gradients. This feature can help the neural network grow much deeper. Principal Component Analysis (PCA) can effectively decrease the sparsity of the data, ensuring that the deep learning model can learn the most important features of data easier. The result of PCA modification can achieve an accuracy of 99%, higher than other works about CSI-based human recognition^9–12^ to the best of my knowledge. This result indicates the fact that preprocessing data might be more crucial than adjusting the structure of the deep learning model if we want to further improve the performance of the deep learning.

### Literature review

Deep learning has dominated machine learning for several years and it can achieve wonderful performance in human motion detection systems. In Ref.^13^, based on the CSI dataset, the authors built a deep learning model, CSI-Net, which can be trained to handle falling detection. The CSI-Net extracts feature of CSI data by the combination of a deconvolutional layer and a convolutional layer. Although the CSI-Net can extract local features, it cannot extract the principal features that can help the deep learning model detect falls better.

The authors in Ref.^14^ designed a deep neural network named WiSPPN, using CSI data for human pose estimation. The WiSPPN works greatly in estimating the human poses through CSI data, but it lacks discussion about how the depth, width, and the structures can affect the performance of the deep learning model in estimating the human poses.

Similarly, Refs.^15,16^, utilized mmWave data to reconstruct human poses. They have achieved great success in reconstructing the human pose through pose estimation. However, the pose estimation from mmWave suffers from the sparse representation, inter-person occlusion^17^, and the decay of mmWave signals. Besides, compared with the human pose estimation by video frames^18,19^, mmWave human pose estimation has a lack of data. The work in Ref.^20^ shows the potential of combining mmWave, red, green and blue (RGB)-D, and Inertial Sensors together for human pose estimation or human activity recognition. However, the applicability of such a multimodal system to real-life scenarios still needs to be tested.

In Ref.^9^, both the RNN-based deep neural network and the CNN-based deep neural network have achieved a great performance in the RGB-D (Red, Green, Blue, and Depth) based Human Motion Recognition. But the author does not mention the influence of data processing and modification of the network structure.

In Ref.^10^, the deep learning model is applied to help the robot to understand human motion. In this work, the authors adapted the structure from the AlexNet^21^ without exploring the effect of structure modifications and data preprocessing. In Ref.^11^, after training the CNN-based network with image-preprocessed radar data, the CNN-based network can outperform the SVM-based approach. Another successful application of deep learning-based human motion detection in the data collected by radar can be found in Ref.^12^, deep learning-based human motion detection can detect walking, falling, sitting, and bending with higher accuracy than the PCA-based scheme. In Ref.^22^, the deep learning model can be applied to high-resolution range information to classify 7 different motions. The deep learning method can gain the highest average classification accuracy in contrast with the PCA-based method and SVM-based method. References^11,12,22^ have proved that the deep learning method can outperform traditional machine learning methods in radar-based human recognition, but they did not experiment with how to further make a progress in the deep learning model.

Overall, it is clear that deep learning can have wonderful performance in many datasets that contain human motion patterns, like CSI data, RGB-D data, and radar data. Besides, deep learning can gain better performance than the previous method, consisting of feature extraction and the traditional machine learning model training. However, these works only implemented the deep learning framework without analysing the effect of deep learning model structure modifications like increasing the width, increasing the depth, and adapting advanced structures in accuracy. They also did not analyze the effect of reducing dimensions on the RF data can have on the performance of the deep learning model. The main contributions of this work are as follows.

- This work focuses on discussing how the model modifications can impact the performance of the deep learning model and how these modifications can be utilized improve the performance in terms of classification accuracy.
- This work considers two aspects of model modification, one is the structure modification, and the other is reducing dimensions.
- After substantial amount of experiments, it has been found that applying LSTM structures and extracting Principle Component Analysis (PCA) features can boost the performance of CSI-based human recognition to a promising accuracy of 99.1%.

## Methods

### Experimental settings

The experimental part composes of two parts. One part is data collection and signal processing and the other part is reconstructing the basic deep learning network.

#### Data collection and signal processing

In this work, we collected samples from sitting and standing at the centre frequency 5.32 GHz. CSI describes how the wireless signals propagate between the RF transmitter and RF receiver and it will perform changes in certain patterns when people perform certain movements, implying the potential of human motion detection. We record the amplitude of CSI using universal software-defined radio peripheral (USRP) when volunteers performed certain human movements (sitting or standing) to construct a dataset of CSI of human activities. USRPs were used because they provide a simple framework that can enable us to collect data easily. Besides, USRPs can transfer and receive frequencies in different bands.

The project uses two USRP devices. One USRP acts as the transmitter and the other acts as the receiver. The device is cabled to two computers and configured to send a signal from one antenna to the other for 10 s. In the experiments to collect CSI data, we kept two USRP devices at a distance of 4 m. Experiments were conducted in an office setting, in the presence of desks, chairs, paintings, etc., simulating real-world situations. Volunteers were asked to complete a sitting and standing action between the two USRPs. When a volunteer acts, the CSI pattern of this action is collected. Specific parameters of the hardware used in this experiment are listed in Table 1:

**Table 1:** Hardware configuration parameters.

Parameters	Values
Platform	USRP X300/X310
Gain (dB)	TX 70, RX 50
TX IP address	192.168.11.1
RX IP address	192.168.10.1
Channel mapping	1 TX, 2 RX
Modulation scheme	QPSK
OFDM subcarriers	64 Subcarriers
Center frequency	5.32 GHz
Interpolation factor	500
Decimation factor	500

**Figure 1:**
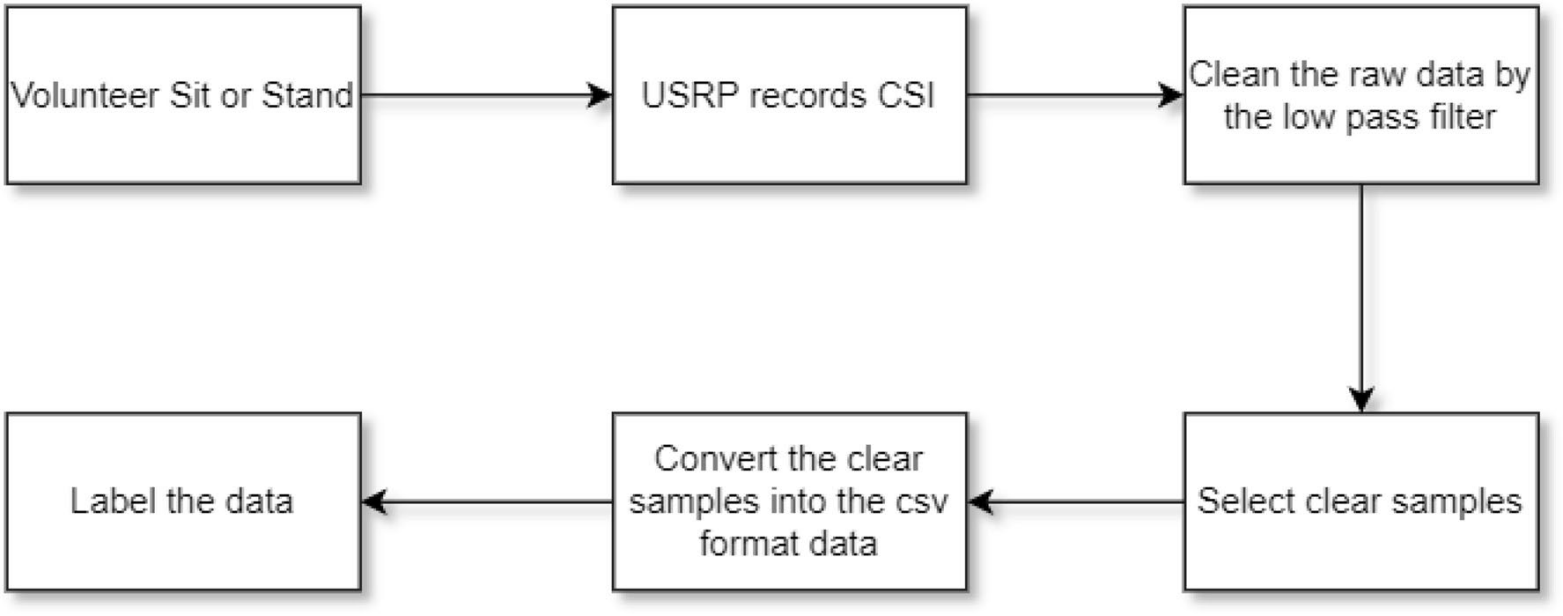
Flow chart of data collection and signal processing.

People do not generally perform sitting and standing activities identically as there will be some variations paired with interference from surrounding factors affecting the CSI. However, the resulting CSI patterns are somewhat similar and correlated to each other. Multiple patterns are recorded from the CSI, which are learned through the deep learning models. To collect data, we asked 10 volunteers to participate. All volunteers signed an ethical approval provided by the Institutional Review Board of the University of Glasgow before the experiment. This process facilitates the collection of CSI for various human movements and creates tags on the CSI samples during collection. After collecting enough sitting and standing samples, the CSI database was successfully constructed. Figure 1 shows the data collection process. The ethical approval to conduct these experiments was obtained by the University of Glasgow’s Research Ethics Committee (approval no.: 300200232, 300190109). All methods were carried out in accordance with the relevant guidelines and regulations and informed consent was obtained from all subjects.

**Figure 2:**
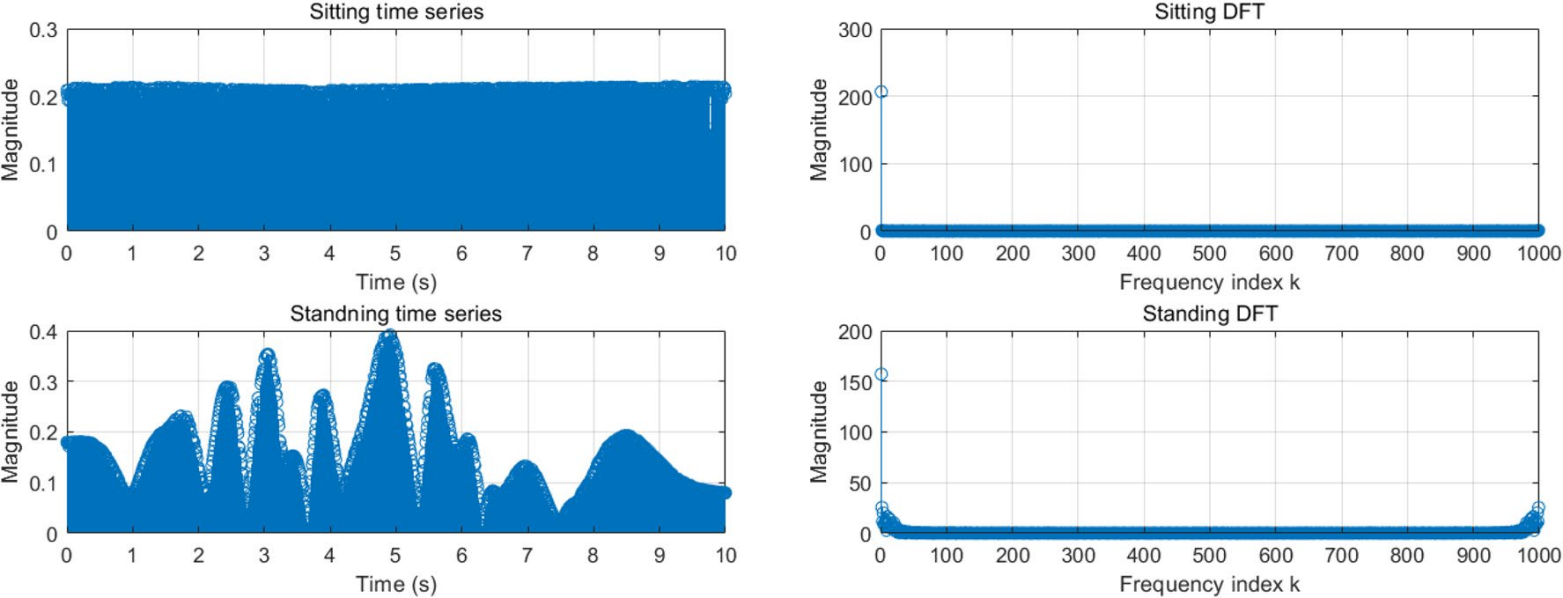
Sitting and standing in time domain and frequency domain.

We initially worked on signal processing to explore the capabilities of CSI and the possibility of using filtering to limit the effects of noise. Specifically, we first analyze the CSI by comparing the sitting and standing CSI in the time and frequency domains using MATLAB programming. Since the acquired data is measured in the time domain, it is displayed directly in the MATLAB graph, while in the frequency domain, the frequency information is displayed using a discrete Fourier transform (DFT). The results of the comparison are shown below in Fig. 2.

It is clear from Fig. 2 that both the sitting and standing noise signals are high frequency. Therefore, a Butterworth low-pass filter was chosen as the noise filter to clean the data. After collecting and processing the data, we move to the deep learning part.

#### Basic neural network

In our previous work^5^, the author constructed a 4-layer multilayer perceptron and each hidden layer has 20 nodes. The structure of this multilayer perceptron can be found in Supplementary Table S1 from the Supplementary Material.

We adopted PyTorch to build the basic neural network. We adopted the Adam^23^ optimizer. The ReLU activation function is used for the hidden layers while the Sigmoid function is applied for the output layer because this task is a binary classifying test.

700 data samples are included in the dataset, 350 data for sitting and 350 data for standing. The train-test validation setting of the dataset is done by randomly splitting 30% of the data as the testing set and 70% of the data as the training set. The train-test split is done by the python library sklearn^24^ with random seeds, and we take an average of 10 times training and testing in each deep learning model to reduce the bias of our results. The mean of 10 times training and testing are reported in Tables 2 and 3.

**Table 2:** Comparison of the original model and structures modified models.

	Accuracy	Precision	Recall	F1-score	Time (ms)
Basic neural network	93.8%	93.7%	95.7%	94.7%	1.36
Basic neural network with ResNet	95.2%	95.2%	95.6%	95.4%	1.23
Deep neural network	68.1%	68.5%	72.2%	70.3%	2.73
Deep neural network with ResNet	97.1%	97.1%	97.2%	97.1%	1.82
Wide neural network	69.0%	69.5%	74.4%	71.8%	4.37
Wide neural network with ResNet	67.6%	68.0%	71.9%	69.9%	7.36
CNN	98.6%	98.6%	98.6%	98.6%	2.30
RNN	95.2%	95.2%	95.4%	95.3%	4.37

**Table 3:** Comparison of structure modified models and PCA models.

	Accuracy	Precision	Recall	F1-Score	Time (ms)
Basic neural network with PCA	98.6%	98.6%	98.6%	98.6%	1.18
Basic neural network with ResNet	95.2%	95.2%	95.6%	95.4%	1.23
Deep neural network with PCA	97.6%	97.6%	97.8%	97.7%	1.03
Deep neural network with ResNet	97.1%	97.1%	97.2%	97.1%	1.82
Wide neural network with PCA	99.0%	99.0%	99.0%	99.0%	2.87
Wide neural network with ResNet	67.6%	68.0%	71.9%	69.9%	7.36
CNN	98.6%	98.6%	98.6%	98.6%	2.30
CNN with PCA	98.6%	98.6%	98.6%	98.6%	0.96
RNN	95.2%	95.2%	95.4%	95.3%	4.37
RNN with PCA	99.1%	99.1%	99.0%	99.0%	2.84
FWCW-ResNet^35^	87.1%	88.2%	89.0%	87.2%	–
5G-enabled^36^	94.6%	95.1%	96.7%	94.6%	–
CNN-LSTM^37^	92.0%	92.3%	92.3%	91.7%	–

In an attempt to assess the performances of those machine learning algorithms, we calculated four classification values, including False Positive (FP), True Positive (TP), False Negative (FN), and True Negative (TN). Then we got the performance metrics based on the above four classification values. The details of calculating performance metrics can be found in equations.

1 $$\begin{aligned} Recall= & {} \frac{TP}{TP+FN}, \end{aligned}$$

2 $$\begin{aligned} Accuracy= & {} \frac{TP+TN}{TP+TN+FP+FN}, \end{aligned}$$

3 $$\begin{aligned} Precision= & {} \frac{TP}{TP+FP}, \end{aligned}$$

4 $$\begin{aligned} F1-score= & {} 2 \times \frac{Precision \cdot Recall}{Precision+Recall}. \end{aligned}$$

**Figure 3:**
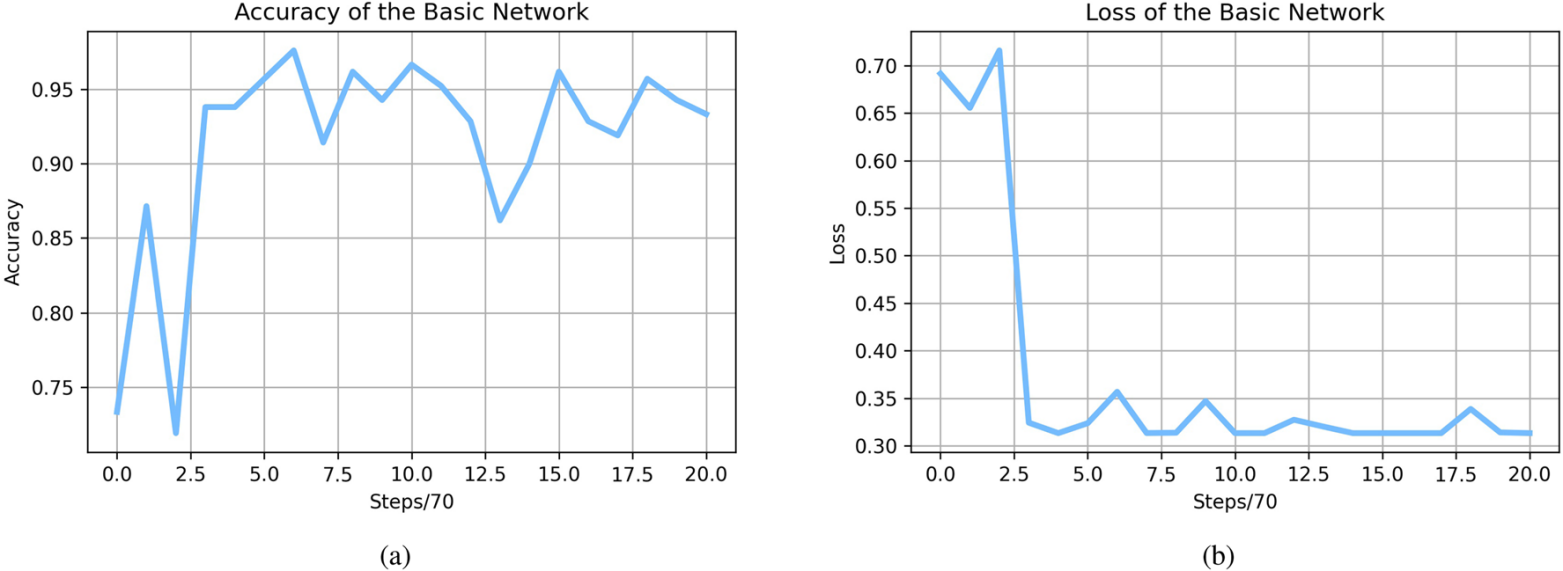
Loss and accuracy of the basic network.

After training and testing 10 times, we get the trained model, which could gain an accuracy of 93.8%, a precision of 93.7%, recall of 95.7%, and an f1-score of 94.7% on average. Compared with the previous work, which got 94.1% in accuracy, 94.3% in precision, 94.1% in recall, and 94.2%, we find that the result of the training is close to the original work. This comparison proved that our reproduction was successful. The loss of accuracy of the basic network is presented in Fig. 3.

The testing accuracy oscillates between 87% and 97% , according to the accuracy curve 3, while the loss rapidly decreases to about 0.32. Test accuracy and loss curves demonstrate that we can train a basic network to converge to an ideal outcome. This fact can demonstrate the effectiveness of training from a different view.

The trained basic network is more accurate at identifying sitting behavior than standing, with a 97% correct prediction rate for standing and a 91% correct prediction rate for sitting, according to the normalized confusion matrix of the basic neural network shown in Supplementary Fig. S1 from the Supplementary Material.

## Structure modifications

### Increase the depth

#### Background

The necessity of increasing the depth originates from the assertion that a neural network can mimic any function. Such assertion is mathematically proved to hold for any function. However, it does not guarantee a computation cost of such approximation. The number of neurons sometimes increases exponentially as the function frequency increases. Thus, the size (i.e. the number of hyper-parameters) explodes quickly. The number of parameters is an important metric in that a large number of parameters usually means a few things. First, it is a lot more costly to train such models computationally. Second, it is more likely to cause under-fitting problems.

The introduction of multi-layered neural networks solves such issues. Properly built multi-layered neural networks should be able to reduce the space complexity of neural networks from exponential to linear. Hence, the problem of parameter exploding is then solved. However, increasing the depth of neural networks introduces other problems too. In the back-propagation phase of training. The gradient accumulates to usually a smaller and smaller number as it propagates backwards because a reasonable learning rate is usually too small for the model to be able to achieve a close approximation towards the global optimally. When approaching the first few layers, gradients tend to be too small to be handled by the floating-point representation. To make this problem even worse, most optimizers have strategies that reduce the magnitude of learning rates as the training progresses. This is because the closer the current state is to the optimal point, the smaller the step length is the better to approach the optimally.

#### Results of increasing the depth

We experimented with changes in the depth of our neural networks and how these changes will influence the result of training. A deep neural network with 12 hidden layers is tested. To test the influence of the depth, all hidden layers share the same number of hidden nodes with the basic neural network, 20 nodes. More details of the deep neural network structure can be found in Supplementary Table S2 from the Supplementary Material. The details of the effects of increasing depth are shown in Supplementary Figs. S3 and S4 of Supplementary Material, without and with PCA, respectively.

An accuracy of 68.1%, a precision of 68.5%, a recall of 72.2%, and an f1-score of 70.3% are obtained by the neural network with a depth of 12, taking average after 10 times training and testing. In comparison to the basic network, there is a significant decrease in all four evaluating metrics.

**Figure 4:**
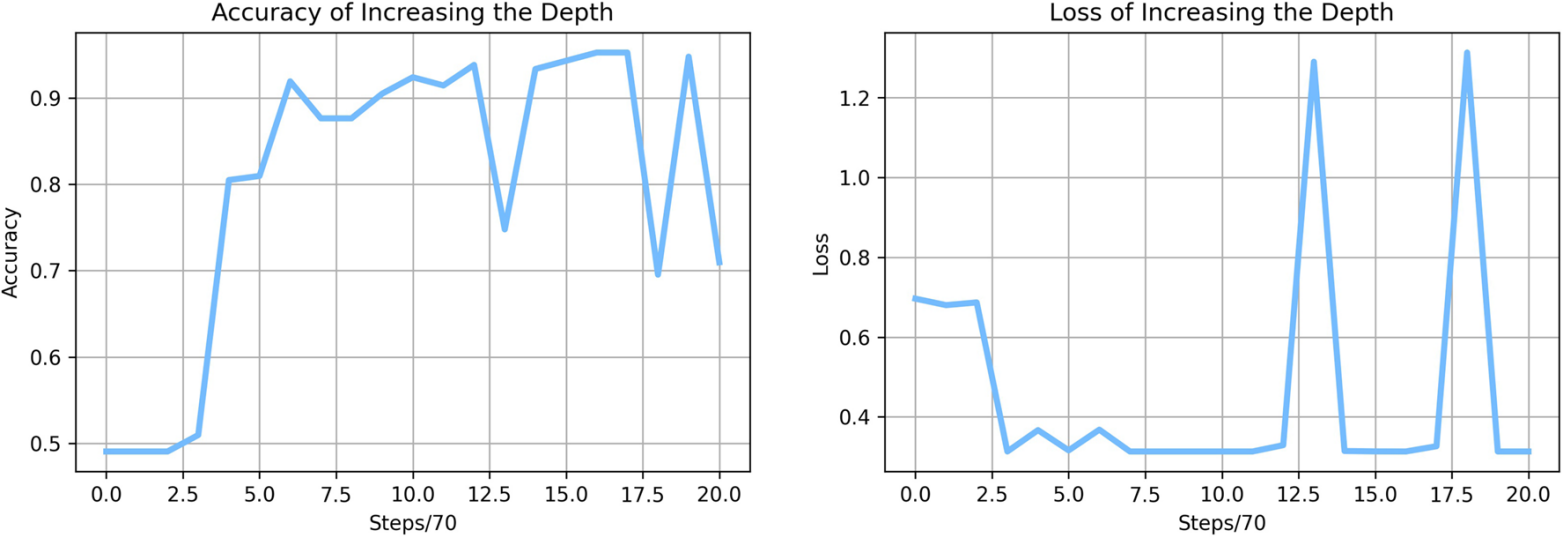
Accuracy and loss of the deep neural network.

When we expand the neural network’s depth to 12, we can see from Fig. 4 that the model’s accuracy first rises to a high level, then oscillates sharply, and finally settles at 68.1% accuracy, which is more than 20% lower than the performance of our simple neural networks. The loss is shown in Fig. 4, which fluctuates between 1.1 and 0.32 on the loss curve in the final stages of training.

This phenomenon of fluctuation in the accuracy and loss curves is expected due to the oscillation nature of deep neural networks.

It is obvious from the deep neural network’s normalized confusion matrix shown in Supplementary Fig. S1 from the Supplementary Material, that it performs poorer at identifying both standing and sitting. The confusion matrix demonstrates that standing is more difficult for the deep neural network to recognize than sitting, with only 62% of standing predictions being true compared to 82% of sitting predictions.

### Increase the width

#### Background

In neural networks, computation is frequently structured into layers of artificial neurons. The layer width refers to the number of neurons in one single layer of the network. There are several reasons why we need a wide neural network.

Firstly, the wide neural network allows each layer to extract a collection of features with more dimensions^25^. If a network is too narrow, each layer can capture a limited dimension of features, there is no possibility that we can extract enough information to propagate down the network, no matter how deep it is. Another reason is the usage of GPU. The GPU is considerably more efficient in parallel calculations on huge tensors, it is more computationally economical^26^ to expand the layers rather than have hundreds of little kernels. Wide residual networks, for example, can compute multiple multiplications in parallel, but deeper residual networks require more sequential calculations (since the computation depends on the previous layer).

As a consequence, we may start with the width to enhance network performance, then increase channel utilization rates at each layer, supplement the thin layer with information from other channels, identify the lowest limit of width, and achieve better performance with as little computing as feasible.

#### Results of increasing the width

To test the effect of width in the neural network, we keep the number of hidden layers as same as that of the basic neural network. At the same time, we increase the number of nodes in each hidden layer from 20 to 320. The parameters of the wide neural network are listed in Supplementary Table S3 from the Supplementary Material.

**Figure 5:**
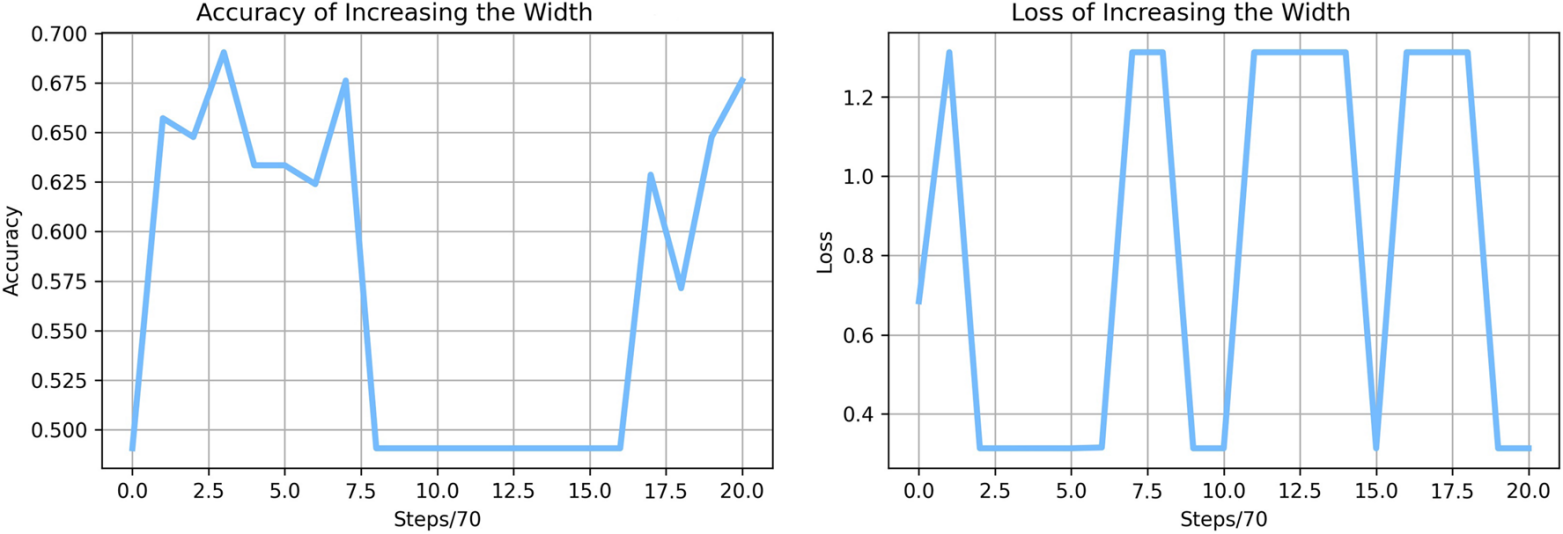
Accuracy and loss of increasing the wide neural network.

While expanding the number of hidden layer nodes to 320 with the same number of hidden layers as the basic network, the accuracy, precision, recall, and f1-score are 69.0%, 69.5%, 74.4%, and 71.8% on average after 10 times training and testing, respectively. Both the accuracy in the testing set and the loss curve in Fig. 5 exhibit significant oscillations. This phenomenon shows that it is difficult to train a wide neural network to distinguish between standing and sitting.

From the normalized confusion matrix of the wide neural network shown in Supplementary Fig. S1 from the Supplementary Material, we can see that making the neural network wide cannot make the neural network classify sitting and standing better. The wide neural network can predict sitting with 86% correct prediction and standing with 62% right prediction. We can find that both the deep neural network and the wide neural network can recognize sitting better than standing while the basic network classifies standing better than sitting from their confusion matrices.

The reason for the bad performance of the wide neural network can be concluded as overfitting: the model attempts to learn too many details from the training data while simultaneously accounting for noise. Overfitting occurs when a model becomes very strong at categorizing or predicting data from the training set but not so good at categorizing data it wasn’t trained on. Adding extra nodes causes overfitting since the original network already fits well with the data.

When we produce a model on training data and use it on the test dataset, the error reduces to a very tiny value, but the error generated from fresh data climbs to a big value, this is an indication of overfitting during training. Because they learn millions or billions of parameters while developing the model, deep neural networks are prone to overfitting. Because it has the potential to do so, a model with this many parameters can overfit the training data. The primary notion behind addressing the problem of overfitting is to reduce the model’s complexity. We may accomplish this by simply eliminating layers or lowering the number of neurons, for example.

The following are some probable causes of overfitting in neural networks: Firstly, the training dataset is rather small. When a network tries to learn from a limited dataset, it tends to have more control over the dataset and ensures that all data points are satisfied exactly. As a result, the network is attempting to memorize every data point while failing to grasp the overall pattern from the training dataset. Secondly, on noisy data, the model tries to make predictions. Overfitting may also happen when a model tries to generate predictions on excessively noisy data, which is produced by an overly complicated model with too many parameters. As a result, the overfitted model is erroneous since the trend does not reflect the reality of the data.

### Advanced neural network structure

#### Background

Recent decades have witnessed great progress in deep learning, new structures of Neural Networks are developed and have been implemented in the field of deep learning with a great outcome. Convolutional Neural Networks, usually named CNN, down-sample inputs to extract features through convolving^27^. Recurrent Neural Networks often referred to as RNN, are known for their ability to handle time-series data^28^. The success of ResNet is a milestone of deep learning that makes the extremely deep neural network possible^29^. Those three new architectures are implemented while keeping the size the same as the neural networks that we experimented with before for a better comparison.

#### CNN

CNN is usually used for image or video recognition where 2D convolutional networks or 3D convolutional networks are implemented. However, since our dataset is only 1D data, we cannot adapt 2D or 3D convolutional networks as people usually do. Instead, we implement a 1D convolutional network and reduce the number of features from 999 to 20 (to keep the size the same as the basic neural network) after the 1D convolutional network. The 1D convolutional network, or feature-extracting network, consists of 2 layers of convolutional networks. Each convolutional neural network in the feature-extracting network is followed by a ReLU function. The rest of the network is the classification network that has four linear hidden layers and one output linear layer with a Sigmoid activation function. Each linear layer has 20 hidden nodes to ensure that the size is as same as the basic neural network. The details of the designed 1D CNN can be found in Supplementary Table S4.

The results after training are shown in Supplementary Fig. S2 from the Supplementary Material.

After training, the accuracy of the CNN achieves 98.6% in the testing set on average. From the above two curves, we can observe that both accuracy and loss become stable at the end of the training, proving that the training of the CNN fits the data well. Compared with the basic network, CNN has a higher testing accuracy (in comparison with 93.8% in the basic neural network) and the training loss is more stable. The precision, recall, and f1-score of CNN are 98.6%, 98.6%, and 98.6%. These results indicate that after extracting features by CNN, the performance of the network can be improved amazingly.

We can learn more about how effective CNN is at categorizing human motion from its normalized confusion matrix, Supplementary Fig. S1 from the Supplementary Material. According to the confusion matrix, the CNN properly distinguishes 98% sitting and 99% standing. When compared to the basic neural network, it is far better (91% and 97%, respectively).

The mathematical principle behind this phenomenon is that the CNN can select the most contributing features through down-sampling while the basic neural network does not have such a strong ability in extracting important features. To be more specific, the CNN selects features by convolving the input data while the basic neural network selects part of inputs through a linear combination that checks all inputs without a focus on a certain part of the input. Some parts of input can be more crucial in determining patterns of input instead of all parts being equally important. So, it is not difficult to infer that CNN, paying attention to the part of inputs, can gain better performance than the basic neural network, treating the whole input as the same.

#### RNN

RNN is specially designed for time-series data as it introduces hidden variables that contain information from the past. To have a better performance in this work, we implement a popular RNN structure, LSTM^30^. Compared with the naïve RNN, LSTM contains three gates (forget gate, input gate, and output gate) that can decide which information to throw away, to store in the cell state, and to output.

The group work of these three gates is the secret behind the wonderful performance of LSTM. In this project, we firstly dropped input size from 999 to 20 through a linear layer, then implemented 4 layers of LSTM, each with 20 hidden nodes, and 1 linear output layer with the Sigmoid activation. The aim is to ensure that the structure of the RNN is as close to the basic network as possible. More details of the RNN can be witnessed in Supplementary Table S5.

Supplementary Figure S2 demonstrates the result of training using an RNN.

After training, we finally get an accuracy of 95.2%, a precision of 95.2%, a recall of 95.4%, and an f1-score of 95.3% in the testing set after 10 times of training and testing. In contrast with the results of the basic network, the RNN can get higher accuracy in the testing set while the training loss is close. Compared with CNN, the curve of loss are more unstable, and the testing set accuracy is a bit lower. This result proves that although RNN can gain a great result in this time-series data, the fact that RNN pays attention to all input instead of part of the input leads to the RNN behaving a little worse than the CNN.

From the normalized confusion matrix of the RNN shown in Supplementary Fig. S1 from Supplementary Material, we can find that the trained basic network is more accurate in recognizing standing behavior than sitting, with 93% correct prediction in sitting while 98% correct prediction in standing.

From the loss curve of the RNN, we can find it converges to 0.3143 at the final stage of training. Unlike the naïve RNN, the LSTM can have a better gradient behavior. The mathematical principles behind this phenomenon are 1. LSTM preserves the error that can be back-propagated through time and layers 2. LSTM contains self-loops where the gradients can flow for a long time.

#### ResNet

The appearance of ResNet made the depth of the Neural Network grow from 19 in VGG^31^ to more than 100. Nowadays, it is common to see a Neural Network with a depth of more than 1000. The problem with the training of the Deep Neural Network is the vanishing gradients. ResNet solves this issue in a clever way, which will be illustrated later. What we do is implement ResNet modification in the basic network, the network with increasing depth, and the network with increasing width, to say how much improvement that ResNet modification can bring to train the basic network, the network with increasing depth, and the network with increasing width respectively.

Supplementary Figure S3 illustrates the results of training the basic network with the ResNet modification. After 10 times training and testing, the testing set accuracy is 95.2%, precision is 95.2%, recall is 95.6% and f1-score is 95.4% on average. From the two curves, we can find that both the training loss and testing set accuracy become smooth at the end of the training. This proves that the training of ResNet is nearly finished. An increase of 1.4% can be found in the testing set accuracy while the change in the training loss is not obvious. This fact proves that the network with the ResNet structure can learn a more intrinsic feature that can help the network distinguish between standing and sitting better.

We can learn more about how much ResNet can help the network at classifying human motion from its normalized confusion matrix shown in Supplementary Fig. S1 from the Supplementary Material. According to the normalized confusion matrix, the basic neural network with ResNet properly distinguishes 92% sitting and 99% standing. When compared to the basic neural network (sitting 91% and standing 97% respectively), it is better in both sitting and standing classification.

Then, we conduct the experiments of ResNet modification in the network with increasing depth with 12 layers. Compared with the original increasing the depth network, an increase of 29.0% can be found in the testing set accuracy (from 68.1 to 97.1%) while the training loss curve is more stable. The amazing increase in testing set accuracy shows the magic that ResNet can bring to the training of the deep network. However, this magic seems to have a side effect when it comes to the network with a larger width (320 nodes in each hidden layer). The wide network with the ResNet modification only acquired an accuracy of 67.6%, a precision of 68.0%, a recall of 71.9%, and an f1-score of 69.9% after training and the training loss oscillates greatly. Compared these measurements with these of the original network, we can find that the wide network with ResNet even performed a little worse than the original wide network.

Compared the normalized confusion matrices of the deep network with ResNet shown in Supplementary Fig. S1 to that of the wide network with ResNet shown in Supplementary Fig. S1, we can find that the deep neural network can classify 96% sitting and 98% standing correctly while the wide neural network can only recognize 82% sitting and 62% sitting.

When the normalized confusion matrices of the deep network with ResNet shown in Supplementary Fig. S1 and the wide network with ResNet shown in Supplementary Fig. S1 are compared, it can be seen that the deep neural network can correctly classify 96% of sitting and 98% of standing while the wide neural network can only identify 82% of sitting and 62% of sitting.

**Figure 6:**
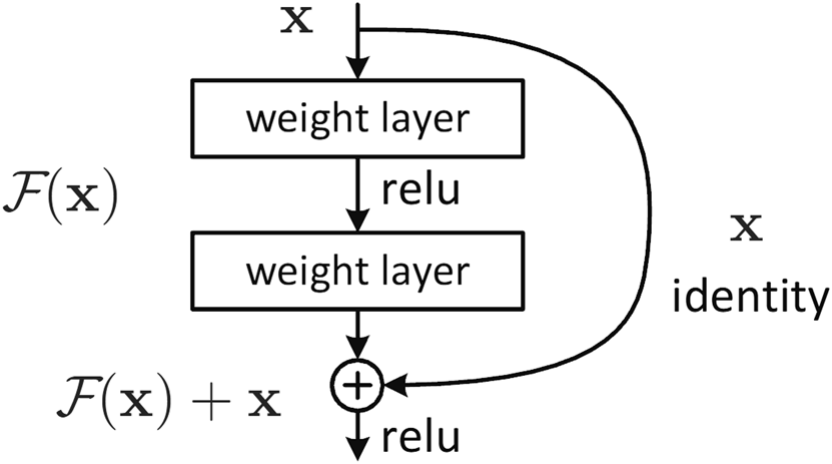
ResNet skip connection structure.

The mathematical principle behind the ResNet is that it successfully transforms the gradients’ backpropagation from multiplicative to additive. This transformation is achieved by the skip connection of the ResNet. The details of the skip connection can be found in Fig. 6.

From its skip connection, the input and the output of the residual block are added, which helps the gradients become additive. The gradient of ResNet can be expressed as:

5 $$\begin{aligned} \frac{\partial E}{\partial x_{l}}=\frac{\partial E}{\partial x_{L}}\left( 1+\frac{\partial }{\partial x_{l}}\sum _{i=1}^{l-1}F(x_{i})\right) . \end{aligned}$$

From this equation, we can find: (1) Any $\frac{\partial E}{\partial x_{L}}$ is directly back-propagated to $\frac{\partial E}{\partial x_{l}}$ plus residual. (2) Since any $\frac{\partial E}{\partial x_{l}}$ is additive rather than multiplicative, it is unlikely to vanish.

Although this modification can solve the problem of vanishing gradient that exists in the deep neural network successfully, it cannot alleviate the issue of overfitting in the wide neural network. Therefore, the deepest network gained the best improvement while the widest network gained the worst.

### Summary of structure modifications

Setting the Basic Neural Network as the baseline, we modified its structure by increasing the depth, increasing the width, applying the CNN, applying the RNN, and applying the ResNet. Next, we trained those modified deep learning models and measured their accuracy, precision, recall, f1-score, and running time when doing the tests in the testing set. We take an average after 10 times training and testing. The results of these measurements are recorded in Table 2.

From the table, we find that CNN, RNN, Basic Neural Network with ResNet, and Deep Neural Network with ResNet can gain better performance than the baseline, while Deep Neural Network, Wide Neural Network, and Wide Neural Network with ResNet perform worse than the baseline if we compare their f1-score.

## Reducing dimensions

### Background

Although the performances of the Deep Neural Network have already been successful after we implement some advanced structures, the original system still suffers from the curse of dimensionality. That means the dimension of wireless signals is too high to have a good performance. To be more specific, as the dimensionality of data rises, the volume of space expands so quickly that the accessible data becomes sparse. Due to this sparsity, the amount of data required to train a deep learning model sometimes climbs amazingly. However, it is difficult to collect more data due to the limitation of the hardware. Therefore, to have a better performance, it is crucial to reduce the dimensionality of the data.

Reducing dimensions can effectively solve the problems of the curse of dimensionality. PCA^32^ is a commonly used technique for reducing dimensions. PCA converts a set of observations of possibly linearly correlated variables into a set of values of linearly uncorrelated variables an orthogonal transformation. Those linearly uncorrelated variables are called principal components. Since the number of principal components should be smaller or equal to the number of original variables, it can be a great unsupervised learning model for reducing dimensions. PCA was firstly proposed by Pearson in 1901^33^, which could only be adapted for non-random variables. Later in 1933, Hotelling^34^ developed PCA for random variables.

In this work, we adapted PCA into our data and reduced the dimension of our data from 999 to 111. A wonderful improvement can be witnessed in the result, illustrated by the following part.

### Results of dimension reduction

Firstly, we implement the data preprocessed by PCA to the Basic Network. Keeping other conditions the same and doing 10 times training and testing, we can achieve an accuracy of 98.6% in the testing set (Supplementary Fig. S4) on average, much higher than the original basic network trained by raw data 93.8%. 98.6% obtained by the Basic Network with PCA is also higher than 95.2% obtained by ResNet in the Basic Network. Besides, both the accuracy curve and the loss curve are more stable at the end of the training, proving the robustness of implementing the PCA into preprocessing.

Next, we do a similar experiment in the 12-layer Neural Network. We can witness a rise of 29.5% in the testing accuracy (see Supplementary Fig. S4). Like the Basic Network case, both the curve of testing accuracy and the curve of loss remain nearly the same at the end of the training process. This result indicates that PCA preprocessing can work well in the deep neural network.

Unlike the ResNet, the magic of PCA preprocessing can still perform well in a wide neural network. The accuracy can be improved from 69.0 to 99.0% after the raw data is preprocessed by PCA while the accuracy obtained by ResNet is 67.6%. Similar to the previous two experiments, the accuracy curve and the loss curve of the 320 Nodes Neural Network become smooth at the end of the training, indicating the Neural Network fitted the data well.

After taking 10 times training and testing, CNN with PCA behaves with little difference compared with CNN without PCA. In contrast, an increase of around 4% can be found in accuracy, precision, recall, and f1-score after RNN taking PCA. The RNN with PCA receives the best result among all models, indicating the best technique for improving the performance of the deep learning model.

According to the normalized confusion matrices of the basic network with PCA, the deep network with PCA, the wide network with PCA, CNN with PCA, and RNN with PCA, we can find that these networks can classify sitting and standing with a correcting rate higher than 95% in the testing set, indicating their amazing abilities in this human motion classification scenario. Apart from our experiments, we added other radio-based deep learning works to compare in the Table 3. The result of comparison shows that LSTM with PCA performs the best, indicating the best technique to improve the performance of deep learning.

From Table 3, we can see that in contrast with the structure modified models, the modification by PCA Dimension Reduction can not only gain better performance in the Basic Network, and the Deep Neural Network but also improve the testing accuracy of the Wide Neural Network, in terms of the testing accuracy.

**Figure 7:**
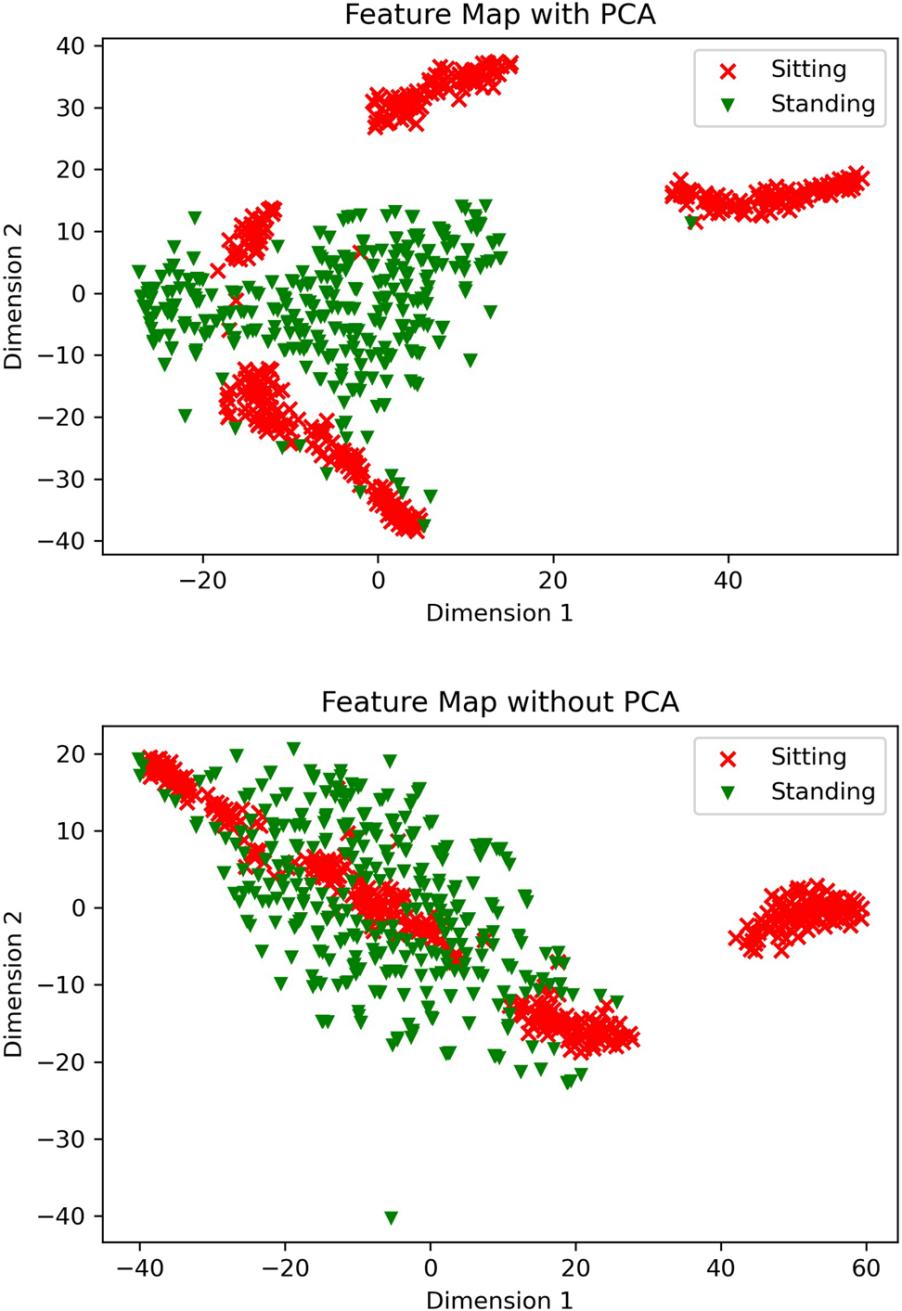
Compare between with PCA Feature Map and without PCA Feature Map.

The secret of the magic of the PCA preprocessing lies in the two-dimensional feature map by t-distributed stochastic neighbor embedding (t-SNE)^38^. Displaying high-dimensional data by assigning a two- or three-dimensional map to each datapoint is the main function of t-SNE. The cooperation between Geoffrey Hinton’s Stochastic Neighbor Embedding and Laurens van der Maaten’s t-distributed variant contributes to the great success of t-SNE in visualizing high-dimensional data. From Fig. 7, we can find: that it is hard to find a boundary between the data points of Sitting and the data points of Standing in the feature map of raw data while it is much easier to draw a boundary between the datapoints of Sitting and the datapoints of Standing after the PCA preprocessing is applied.

The reason that low dimensional data has better performance is that we extract principle components of the raw data by PCA, the influences of noise and high dimensions are removed, and it becomes much easier for the deep learning model to learn how to classify Sitting and Standing. Although ResNet can solve the problem of vanishing gradients, it cannot perform well if the data is hard to classify due to the influence of noise and high dimensions. Therefore, reducing dimensions should be an important part of the training the deep learning model.

## Discussion and conclusion

In this work, we first collect a CSI dataset of sitting and standing activities and build a basic network to classify these activities using PyTorch.

Thereafter, we increase the depth and the width of the neural network, adopt advanced neural network structures, and reduce dimensions by PCA to observe how the testing accuracy and loss curve will change. Through these changes, we thoroughly explore how to modify the deep learning in this wireless CSI dataset.

Although increasing the depth can improve the expressiveness of the neural network and extract abstract features of the input, problems of vanishing gradients can become more serious as the depth increases. This problem leads to slow training and a bad result in both the testing accuracy and the loss. Increasing the width can increase the dimensions of the feature map in the hidden layers. However, too many parameters in the hidden layers can lead to the problem of overfitting.

Transforming the structure of the basic network to CNN can have an amazing improvement in the result as CNN can sample the most important features to improve the training efficiency of the classifying network. RNN can store the past information in its hidden variables, and LSTM improves the naive RNN by introducing the input gate, the forget gate and the output gate. Although LSTM is specially designed for time-series data, it focuses on all input instead of the most important features like CNN. Therefore, it performs a little worse than CNN.

ResNet is a milestone of deep learning that makes the extremely deep neural network possible. From our experiments, we can find that the ResNet can increase the testing accuracy of a 12-layer neural network with 20 nodes in each hidden layer by 29% while it cannot work well for a 4-layer neural network with 320 nodes in each hidden layer. Therefore, ResNet is effective for a deep neural network, but it cannot be so effective for a wide neural network. In comparison with ResNet, reducing dimensions by PCA can increase the testing accuracy of the Basic Network, the testing accuracy of the 12-layer neural network with 20 nodes in each hidden layer, and the testing accuracy of the 4-layer neural network with 320 nodes in each hidden layer all to around 98%. CNN with PCA has not much difference while RNN with PCA improves accuracy, precision, recall, and f1-score to around 99%.

The highest accuracy is gained by the combination of RNN and PCA, with an accuracy of 99.1%. In Ref.^39^, semi-supervised training was applied radar-based Human Activity Recognition with an accuracy of 87.6%. The article^35^ applied ResNet^29^ to recognize human activities with an overall accuracy of 96%. While in Ref.^40^, the team designed the HARnet for mmWave based real-time human activity recognition. HARnet can achieve an off-line accuracy of 93.25% and real-time accuracy of 91.52%. According to Ref.^41^, a hybird framework composed by 1-D Convolutional Neural Network, Recurrent Neural Network, and 2-D Convolutional Neural Network is implemented for radar-based human activity recognition with an accuracy around 95%. In Ref.^42^, five distributed pulsed Ultra-Wideband (UWB) radars in a coordinated network is applied to human activity recognition, bringing an average improvement of 17.5%. 1D-CNN applied in Ref.^36^ gained an accuracy 94.60% while^37^ applied a multi-view CNN-LSTM, resulting in an accuracy of 92.00%. Although these recent works have a great performance in radar-based human activity recognition, they still do not surpass the accuracy of our method through combining LSTM and PCA (99.1%).

The training processing of PCA preprocessed data is more stable compared with the ResNet. The feature map of the raw data and the feature map of the PCA preprocessed data shown in Fig. 7 shows that reducing dimensions by PCA can help the CSI data become easier to classify.

In conclusion, modifying the neural network Structure and reducing dimensions can be two perspectives in improving the deep learning model for the contactless AI-enabled human motion detection system. Reducing dimensions might play a more crucial role in the improvement.

## Supplementary Information

Supplementary Information.

## Data Availability

The datasets used and analysed during the current study are available from the corresponding author on reasonable request.
