# Supplementary material for: Evaluation of deep learning models in contactless human motion detection system for next generation healthcare
Source: Sci Rep. 2022 Dec 14;12:21592. doi: 10.1038/s41598-022-25403-y (PMC9751145; doi:10.1038/s41598-022-25403-y)
Supplement: Supplementary file 1 — Supplementary Information. [file 41598_2022_25403_MOESM1_ESM.pdf]

5 Supplementary Material

In this section, we list structures of neural networks, confusion matrices of neural networks, and accuracy and loss curves for readers who want to check more details of the results of our work.

Layers	Type of Layers	Size of Input	Number of Nodes	Size of Output
Input Layer	Linear Layer	999×1	999	20×1
Hidden Layer1	Linear Layer	20×1	20	20×1
Hidden Layer 2	Linear Layer	20×1	20	20×1
Hidden Layer 3	Linear Layer	20×1	20	20×1
Hidden Layer 4	Linear Layer	20×1	20	20×1
Output Layer	Linear Layer	20×1	2	2×1

Table 4. Structure of the Basic Network

Layers	Type of Layers	Size of Input	Number of Nodes	Size of Output
Input Layer	Linear Layer	999×1	999	20×1
Hidden Layer1	Linear Layer	20×1	20	20×1
Hidden Layer 2	Linear Layer	20×1	20	20×1
Hidden Layer 3	Linear Layer	20×1	20	20×1
Hidden Layer 4	Linear Layer	20×1	20	20×1
Hidden Layer 5	Linear Layer	20×1	20	20×1
Hidden Layer 6	Linear Layer	20×1	20	20×1
Hidden Layer 7	Linear Layer	20×1	20	20×1
Hidden Layer 8	Linear Layer	20×1	20	20×1
Hidden Layer 9	Linear Layer	20×1	20	20×1
Hidden Layer 10	Linear Layer	20×1	20	20×1
Hidden Layer 11	Linear Layer	20×1	20	20×1
Hidden Layer 12	Linear Layer	20×1	20	20×1
Output Layer	Linear Layer	20×1	2	2×1

Table 5. Structure of the Deep Neural Network

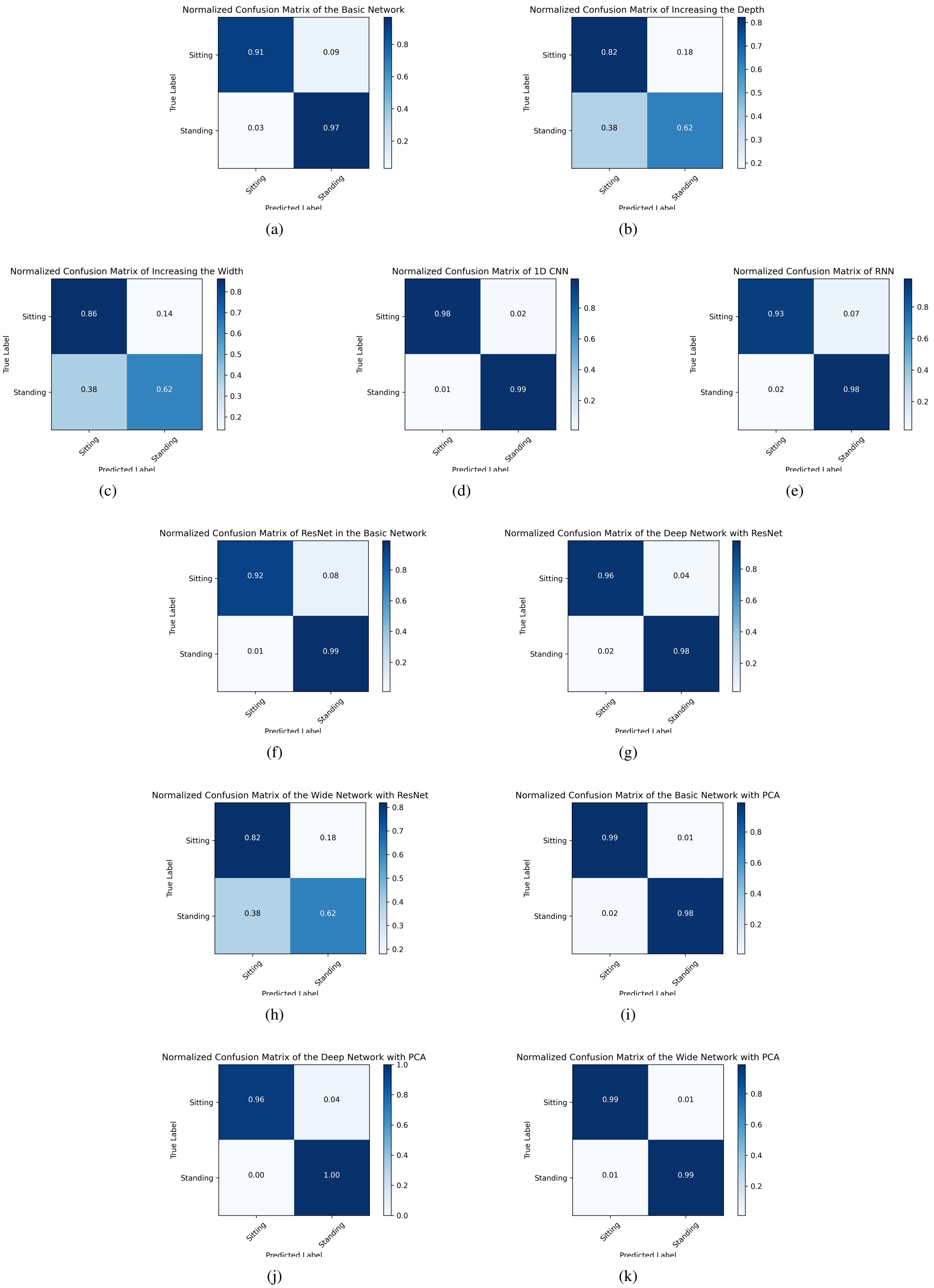

Figure 8. Normalized Confusion Matrix

Layers	Type of Layers	Size of Input	Number of Nodes	Size of Output
Input Layer	Linear Layer	999×1	999	320×1
Hidden Layer1	Linear Layer	320×1	320	320×1
Hidden Layer 2	Linear Layer	320×1	320	320×1
Hidden Layer 3	Linear Layer	320×1	320	320×1
Hidden Layer 4	Linear Layer	320×1	320	320×1
Output Layer	Linear Layer	320×1	2	2×1

Table 6. Structure of the Wide Neural Network

Layers	Type of Layers	Size of Input	Number of Kernels	Kernel Size	Stride	Size of Output
Feature Extraction Layer 1	Convolution Layer	999×1	1	9	9	111×1
Feature Extraction Layer 2	Convolution Layer	111×1	1	16	5	20×1
Classification Layer 1	Linear Layer	20×1	-	-	-	20×1
Classification Layer 2	Linear Layer	20×1	-	-	-	20×1
Classification Layer 3	Linear Layer	20×1	-	-	-	20×1
Classification Layer 4	Linear Layer	20×1	-	-	-	20×1
Output Layer	Linear Layer	20×1	-	-	-	2×1

Table 7. Structure of the CNN

Layers	Type of Layers	Size of Input	Number of Nodes	Size of Output
Input Layer	Linear Layer	999×1	999	20×1
Hidden Layer1	LSTM	20×1	20	20×1
Hidden Layer 2	LSTM	20×1	20	20×1
Hidden Layer 3	LSTM	20×1	20	20×1
Hidden Layer 4	LSTM	20×1	20	20×1
Output Layer	Linear Layer	20×1	2	2×1

Table 8. Structure of the RNN

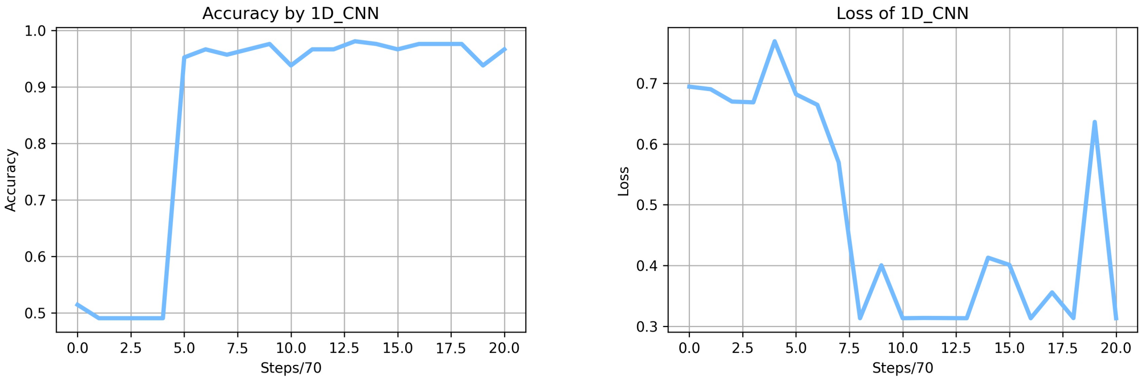

(a)

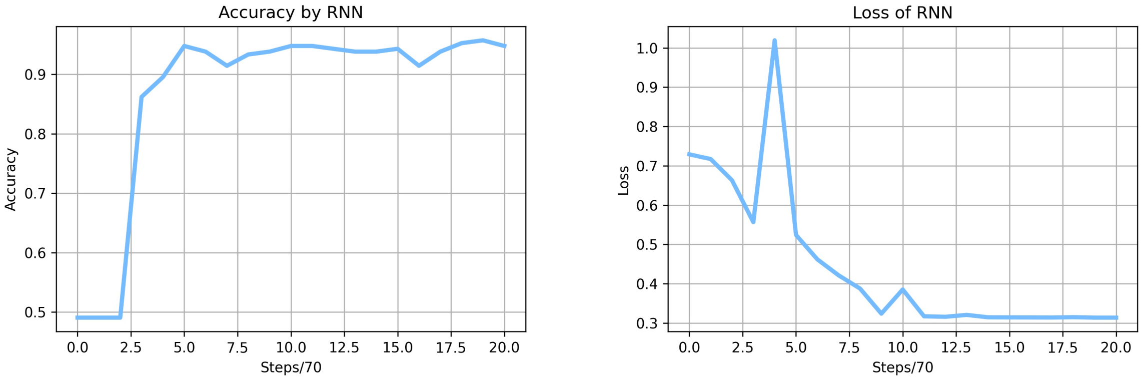

(b)

Figure 9. Accuracy and Loss of CNN and RNN

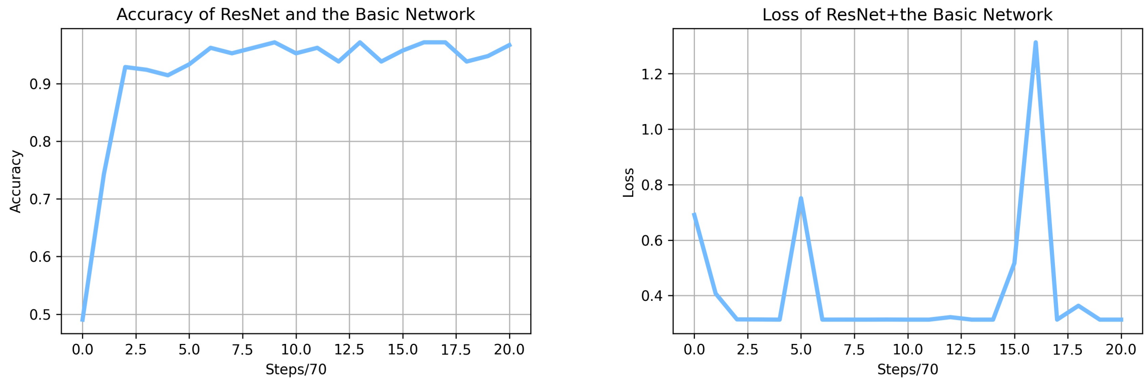

(a)

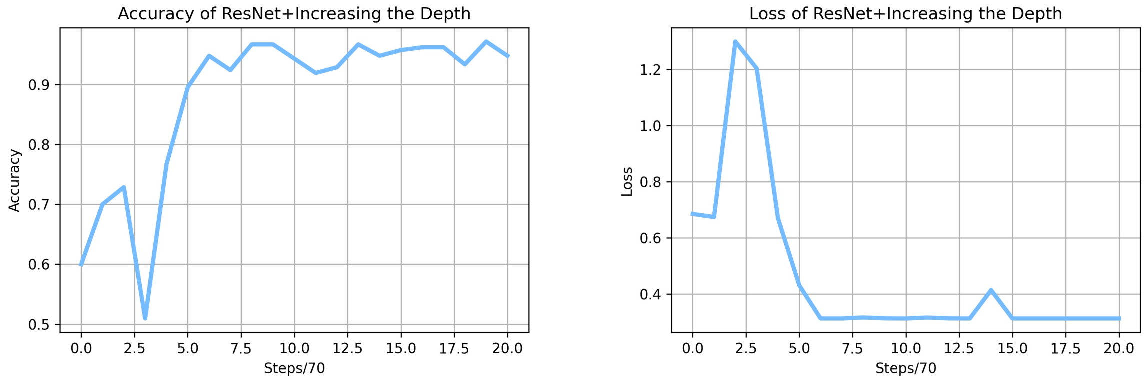

(b)

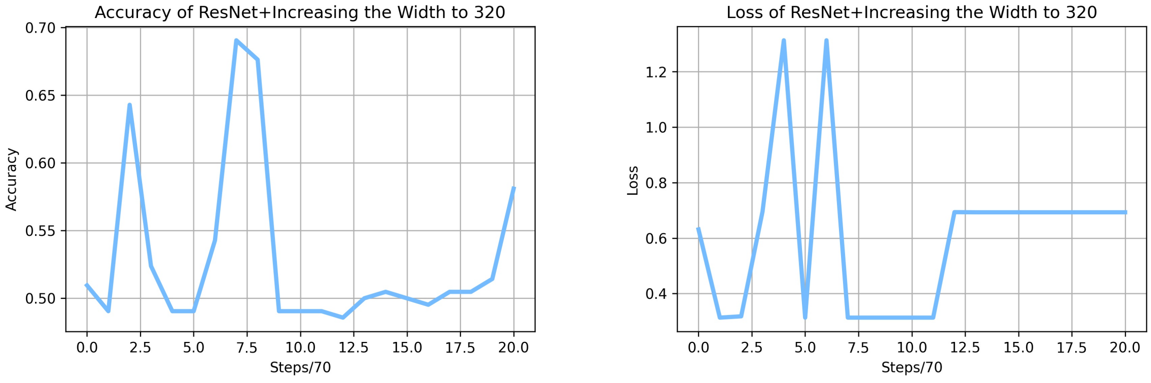

(c)

Figure 10. Accuracy and Loss of ResNet

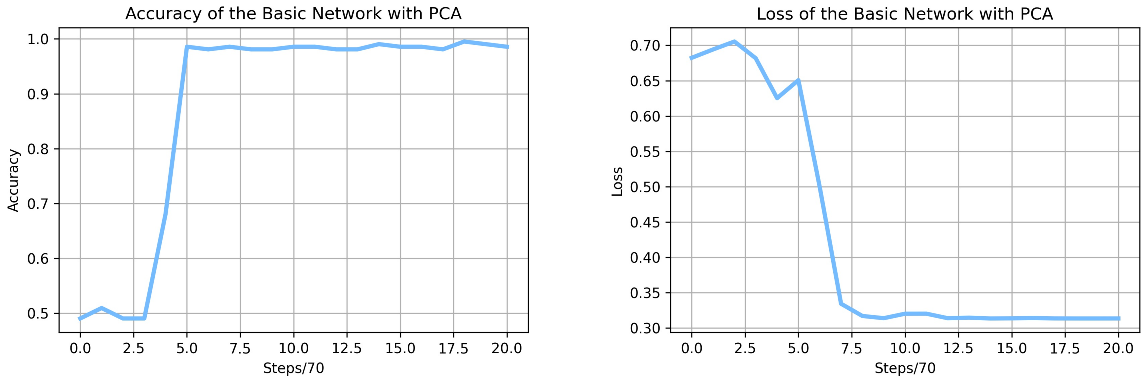

(a)

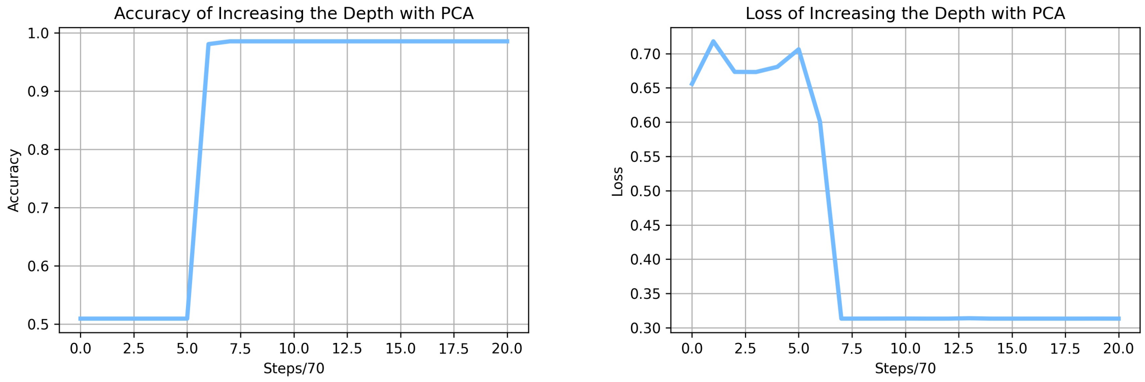

(b)

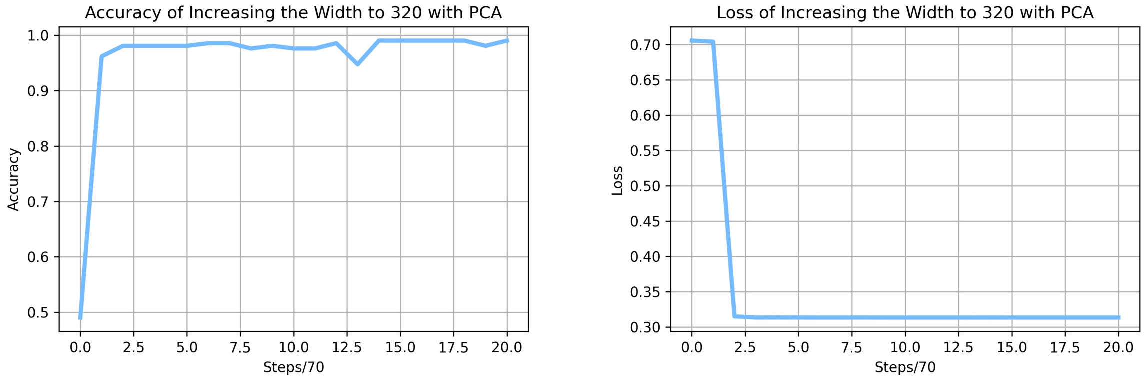

(c)

Figure 11. Accuracy and Loss of Networks with PCA
